# Factors affecting cognitive frailty improvement and progression in Taiwanese older adults

**DOI:** 10.1186/s12877-024-04700-3

**Published:** 2024-01-29

**Authors:** Lalu Suprawesta, Sy-Jou Chen, Hui-Yu Liang, Hei-Fen Hwang, Wen-Yu Yu, Mau-Roung Lin

**Affiliations:** 1 Institute of Injury Prevention and Control, College of Public Health, Taipei Medical University, 250 Wu-Hsing Street, Taipei, 11031 Taiwan, ROC; 2 Department of Sport and Health Education, Faculty of Sport Science and Public Health, Universitas Pendidikan Mandalika, Mataram, West Nusa Tenggara Indonesia; 3 Department of Emergency Medicine, Tri-Service General Hospital, National Defense Medical Center, Taipei, Taiwan, ROC; 4 Department of Nursing, National Taipei University of Nursing and Health Sciences, Taipei, Taiwan, ROC; 5 Department of Emergency Medicine, Taipei Medical University Hospital, Taipei, Taiwan, ROC; 6 Programs in Medical Neuroscience, College of Medical Science and Technology, Taipei Medical University, Taipei, Taiwan, ROC

**Keywords:** Cognitive frailty, Older adults, Risk factor, Trajectory, Taiwan

## Abstract

**Background:**

Knowledge of predictors of cognitive frailty (CF) trajectories is required to develop preventive strategies to delay or reverse the progression from CF to dementia and other adverse outcomes. This 2-year prospective study aimed to investigate factors affecting the progression and improvement of CF in older Taiwanese adults.

**Methods:**

In total, 832 community-dwelling people aged ≥ 65 years were eligible. Fried’s five frailty criteria were used to measure prefrailty and frailty, while cognitive performance was assessed by the Clinical Dementia Rating and Mini-Mental State Examination. Each component of reversible CF and potentially reversible CF was assigned a score, with a total score ranging 0 to 5 points. Two annual follow-up CF assessments were conducted. The group-based trajectory model was applied to identify latent CF trajectory groups, and a multinomial logistic regression was used to examine relationships of explanatory variables with CF trajectories.

**Results:**

According to data on 482 subjects who completed the two annual follow-ups, three CF trajectories of robust, improvement, and progression were identified. After adjusting for the baseline CF state, CF progression was significantly associated with an older age (odds ratio [OR] = 1.08; 95% confidence interval [CI], 1.02 ~ 1.14), a lower Tinetti balance score (OR = 0.72; 95% CI, 0.54 ~ 0.96), a slower gait (OR = 0.98; 95% CI, 0.97 ~ 0.99), and four or more comorbidities (OR = 2.65; 95% CI, 1.19 ~ 5.90), while CF improvement was not significantly associated with any variable except the baseline CF state. In contrast, without adjusting for the baseline CF state, CF progression was significantly associated with an older age, female sex, balance scores, gait velocity, regular exercise, the number of comorbidities, and depression, while CF improvement was significantly associated with female sex, balance scores, and the number of comorbidities.

**Conclusions:**

The baseline CF state, an older age, poorer balance, slower gait, and a high number of comorbidities may contribute to CF progression, while the baseline CF state may account for associations of engaging in regular exercise and depression with CF development.

**Supplementary Information:**

The online version contains supplementary material available at 10.1186/s12877-024-04700-3.

## Introduction

Cognitive frailty (CF) is the simultaneous presence of physical frailty and cognitive impairment, excluding the presence of dementia, Parkinson’s disease, or other neurodegenerative diseases [1]. Relative to physical frailty or cognitive impairment alone, CF increases the risk of adverse health outcomes, such as falls, dementia, hospitalizations, disabilities, and all-cause mortality [2–4], thus emphasizing the importance of targeting CF in reducing adverse health outcomes and their social costs.

The prevalence of CF, as assessed by a definition of combining physical frailty and mild cognitive impairment (MCI) [1], has increased over the past decade and was estimated to be 6% in 2012 to 2017 and 11% in 2018 to 2020 in the world [5]. The CF prevalence among older Taiwanese adults is between 8.6% and 13.3% [6, 7]. Since the predictive value or the number of identified cases is strongly affected by disease prevalence in a population, the low prevalence of CF suggests the limited clinical utility of identifying CF cases for early interventions to prevent adverse health outcomes [8]. In other words, as the prevalence of CF increases through other operational definitions, the identified number of CF cases may also increase for healthcare interventions in an efficient way [9]. Recently, CF was defined through another definition, and two subtypes were suggested: reversible CF (RCF) and potentially RCF (PRCF), in which RCF is characterized by pre-frailty or frailty and subjective cognitive decline (SCD) and PRCF is characterized by pre-frailty or frailty and MCI [10]. Both RCF and PRCF may predict progression to dementia [11, 12]. Importantly, the high prevalence of RCF (e.g., 27.8% in an older Chinese population with intact cognition [13]) may also improve the predictive power of pre-frailty for adverse health outcomes [14] in that relative to the original CF definition, older adults based on the RCF and PRCF definitions can be efficiently targeted for prevention of dementia and other adverse outcomes at an early time.

Prior studies have reported that risk factors of CF may include an older age, female sex, low educational level, low physical or cognitive activities, low vitamin D, low high-density lipoprotein (HDL) cholesterol, having eye problems, functional mobility, a slower gait velocity, greater double-support time variability, multimorbidities, global cognition, processing speed, depression, balance confidence, and life satisfaction [15–20]. However, causal inferences of these risk factors for CF are limited because most of these findings are from cross-sectional studies. Furthermore, a risk factor for CF identified from a cohort study may not be stable in predicting the occurrence of CF over time because CF status is changeable within a short time period and a considerable of older adults were observed to have distinct CF states in two adjacent years [20]. To our knowledge, no study has reported longitudinal paths of CF (i.e., CF trajectories over time) and their affecting factors. A developmental trajectory on the basis of clusters of individual characteristics describes the persistent course of an observed outcome (e.g., CF) over time, so that affecting factors of the trajectory are stable over time [21]. Knowledge of changes in CF states and predictors of CF trajectories is required to develop preventive strategies effectively to delay or reverse the progression from CF to dementia; however, there is still a lack of the information in the literature.

Accordingly, a longitudinal cohort study was conducted to investigate factors affecting CF trajectories and additionally describe transitions of CF states among older Taiwanese adults living in the community over a 2-year period.

## Methods

### Study participants

Eligible participants aged ≥ 65 years, who could independently ambulate and were community-dwelling in the metropolitan Xinyi District of Taipei City, were enrolled in August 2017 to June 2019 from outpatient clinics at Taipei Medical University (TMU) Hospital (Taipei, Taiwan). Individuals who had difficulty performing basic daily tasks, had communication difficulties, or had a major disease (e.g., advanced cancer, a major cardiovascular disease, or dementia) were excluded, according to the baseline data and their medical history. The Institutional Review Board of TMU approved the protocol of this study, and written informed consent was obtained from each participant prior to enrollment.

**Fig. 1:**
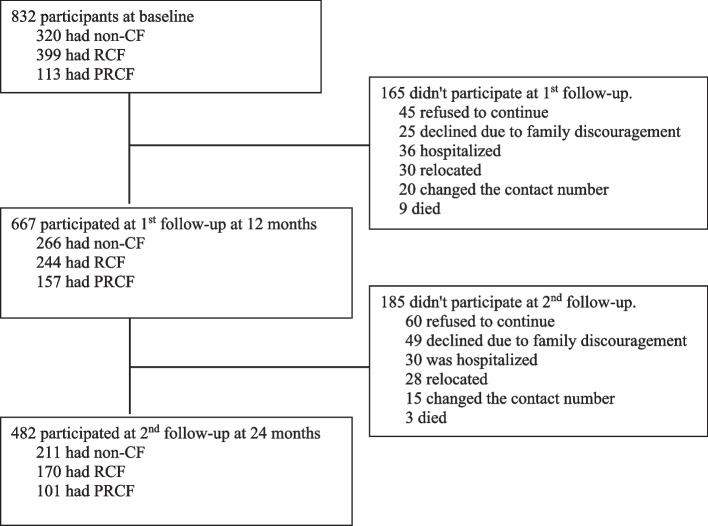
Flow diagram of participants with three cognitive frailty (CF) states of non-CF, reversible CF (RCF), and potentially RCF (PRCF) at the baseline and two annual follow-up assessments

Of 832 individuals who participated in the baseline assessment, 667 completed the first follow-up and 482 completed two follow-ups. The reasons that subjects declined or were unable to attend the two follow-ups were poor health, family issues (e.g., taking care of a sick spouse or grandchildren), weather conditions (e.g., extreme temperatures or heavy rain), and the need to accompany a family member. A flow diagram of participants at the baseline and two follow-up assessments is shown in Fig. 1.

### Data collection

At the baseline and two follow-ups at 12 and 24 months, data on CF states were assessed. According to prior studies [15–19], data on sociodemographic and health-related characteristics, and functional and gait measures that potentially are CF predictors were collected.

### Cognitive-frailty assessment and scoring

In this study, CF was defined as a heterogeneous clinical syndrome combining cognitive impairment and pre-frailty or frailty and was excluded if there was dementia resulting from Alzheimer’s disease (AD) or other conditions [10]. To assess cognitive impairment, MCI was determined by the Clinical Dementia Rating (CDR) [1]. CDR scores of 0, 0.5, and ≥ 1 were used to indicate no dementia, MCI, and dementia, respectively [22]. Furthermore, the SCD status was indicated by a positive response to a self-reported persistent decline in cognitive capacity compared to a previously normal cognitive status during the previous 2 years [23], with no evidence of objective cognitive impairment (CDR = 0 in this study).

Frailty states were assessed by phenotype frailty criteria comprised of five components of weight loss, weakness, exhaustion, low physical activity, and slowness [24]. Unintentional weight loss was defined as > 3 kg or 5% of body weight over the past year. Weakness was assessed by grip strength of the right hand using a handgrip dynamometer. Low grip strength was sex-specifically defined at ≤ 29 kg for males and ≤ 17 kg for females [24]. Exhaustion was determined as a positive response to an item (“I feel that everything I do is an effort”). Slowness was defined as a gait velocity of < 0.8 m/s at a normal walking pace. Physical activity was quantified using the International Physical Activity Questionnaire-Short Form [25], and low physical activity was defined as having fewer than 3 days of vigorous-intensity activity for at least 20 min per day or fewer than 5 days of moderate-intensity activity or walking for at least 30 min per day [24]. Overall, the presence of three or more of the five components was considered to indicate frailty, the presence of one or two components as pre-frailty, and the absence of all five components as non-frailty.

In this study, CF consisted of two subtypes, in which RCF was defined as the presence of both pre-frailty/frailty and SCD, while PRCF was defined as the presence of pre-frailty/frailty and MCI [10]. Non-CF was indicated when a subject had pre-frailty/frailty with normal cognitive function (CDR = 0 and no SCD) or had SCD/MCI with non-frailty. To quantify complex changes in CF states from the baseline to each follow-up assessment, each of the RCF and PRCF components was assigned a score. For CF scoring, the presence of pre-frailty or SCD only was assigned 1 point; the presence of frailty or MCI only, 2 points; the simultaneous presence of pre-frailty and SCD, 3 points; the simultaneous presence of pre-frailty and MCI or frailty and SCD, 4 points; and the simultaneous presence of frailty and MCI, 5 points (see Supplementary Table 1). A non-CF state was assigned 0 points.

### Sociodemographic and health-related characteristics

Sociodemographics and lifestyle behaviors consisted of age, sex, body-mass index (BMI), educational level, monthly household income, regular exercise habits, current smoking, and current alcohol consumption. The BMI was calculated as the weight (kg) divided by height squared (m^2^), and participants were categorized as underweight (< 18.5 kg/m^2^), normal weight (18.5 ~ 22.9 kg/m^2^), overweight (23 ~ 24.9 kg/m^2^), and obese (≥ 25 kg/m^2^) [26]. Health-related characteristics consisted of preexisting comorbidities and medications, as well as depressive symptoms. Preexisting comorbidities were assessed using a list of 12 chronic conditions (hypertension, diabetes, heart disease, malignant tumors, respiratory tract diseases, arthritis or rheumatism, gastric ulcers, liver diseases, cataracts, kidney diseases, gout, and spinal spurs). Medications for these chronic conditions were documented. Additionally, depressive symptoms were assessed using the 15-item Geriatric Depression Scale (GDS), with a score of > 5 assessed as being indicative of depression [27].

### Functional and gait measures

Gait characteristics were assessed using the 6-m GAITRite electronic walkway (CIR Systems, Franklin, NJ, USA), where participants were asked to walk on a walkway at their usual pace. Eight temporal and spatial gait characteristics of velocity (cm/s), cadence (steps/min), step width (cm), stride length (cm), stride length variability (%), stride time variability (%), swing time variability (%), and double-support time variability (%) were measured in this study. Variability was expressed using a coefficient of variation as a ratio of the standard deviation (SD) to the mean multiplied by 100. Tinetti’s gait test consists of nine gait maneuvers, and the score ranges 0 to 13 points, with a higher score indicating better mobility, while Tinetti’s balance test consists of 13 static and dynamic balance maneuvers, and the score ranges 0 to 24 points, with a higher score indicating better balance ability [28]. The Older Adults Resources and Services (OARS) activities of daily living (ADLs) scale assesses seven basic ADLs and seven instrumental ADLs [29]. The ADL score ranges 0 to 28, with a higher score indicating greater physical independence.

The activities-specific balance confidence (ABC) scale assesses an individual’s confidence in performing 16 common daily tasks without losing their balance [30, 31]. The ABC score ranges 0 to 100, with lower scores indicating greater balance confidence. The Mini-Mental State Examination (MMSE), which comprises six domains of orientation, registration, recall of information, attention and calculation, language, and visuospatial construction, was used to assess global cognitive function. The total MMSE score ranges from 0 to 30, with a higher score indicating better global cognitive function [32, 33].

### Statistical analysis

Descriptive statistics are reported as the mean and SD for continuous variables and as an absolute number and percentage for categorical variables. Transitions of the three CF states (non-CF, RCF, and PRCF) at the 2-year follow-up from the baseline assessment for men and women were calculated, and distributions of CF transitions between men and women were compared using Pearson’s Chi-squared test. Distributions of score changes of CF at the 2-year follow-up among baseline characteristics were tested using Student’s *t*-test or an analysis of variance (ANOVA) test, and a binary logistic regression model was used to compare differences in baseline characteristics between participants who completed the follow-up assessments and those who did not.

The group-based trajectory model was applied to identify latent trajectory groups in the study population [34]. While the model presumes that the study population was composed of distinct subpopulations that were not identifiable based on observed characteristics, the number of CF trajectory groups that represent heterogeneity in a study population is usually determined using the criteria of the lowest value of Bayesian information criteria and an average posterior probability of group assignments of ≥ 0.70. According to the CF score assigned to each participant at each time point, we specified and compared four nested models (i.e., assuming one to four latent trajectory groups); in consequence, the model with three CF trajectories, i.e., robust (stable in CF scores), improvement (negative changes in CF scores), and progression (positive changes in CF scores) was selected. To examine whether features of the three CF trajectories were reliable, a sensitivity analysis with four nested group-based trajectory models was also conducted for 482 participants who completed both follow-up assessments; consequently, the model with three CF trajectories had the lowest value of Bayesian information criteria.

Among the three CF trajectory groups, baseline characteristics were compared using an ANOVA or Pearson’s Chi-squared test. A multinomial logistic regression model was applied to investigate independent associations of explanatory variables with improvement and progression of CF compared to the robust group. In the bivariable multinomial logistic regression analysis, explanatory variables with *p* < 0.20 were selected for the initial multivariable analysis to avoid large type-II errors in variable selection [35], and for selecting variables in the final multivariable analysis, the level of statistical significance was set to *p* < 0.05, with the exception of age and sex that were retained in the final model because of their biological and clinical importance to the frailty state and cognitive function. In statistical modeling, when the value of the baseline CF state was 0 counts, we replaced that with 1 to resolve the problem of non-convergence in the logistic regression. All these data analyses were performed using SPSS vers. 25.0 for Windows (IBM, Armonk, NY, USA) or SAS vers. 9.4 (SAS Institute, Cary, NC, USA).

## Results

Of 832 eligible participants at the baseline, 320 (38.5%) exhibited non-CF, 399 (47.9%) had RCF, and 113 (13.6%) had PRCF. As Fig. 1 shows, 482 subjects completed two follow-up assessments, of which 211 (43.8%) had not developed CF, 170 (35.3%) had developed RCF, and 101 (20.9%) had developed PRCF by the second follow-up. According to results of the binary logistic regression analysis, compared to participants who completed the two follow-ups, those who did not complete the follow-ups were significantly more likely to have had RCF (odds ratio [OR] = 1.45) and PRCF (OR = 1.87) at the baseline, to be a current smoker (OR = 1.87), to have lower scores of Tinetti’s balance (OR = 0.83), to have larger variability in the stride length (OR = 1.11), and to have smaller variability of double-support time (OR = 0.96).

Table 1 shows the CF transitions at the second follow-up from the baseline among 160 men and 322 women. For the baseline CF state, women were significantly more likely to have had RCF and PRCF compared to men. On the other hand, for the CF transitions, no significant differences between men and women from the baseline non-CF (*p* = 0.929) and baseline PRCF (*p* = 0.101) were detected, while a significant difference from the baseline RCF (*p* = 0.024) was detected.

**Table 1:** Transition of three cognitive frailty (CF) states of non-CF, reversible CF (RCF), and potentially RCF (PRCF) from the baseline to the second follow-up assessment in men and women

Transitional CF state	All (*N* = 482) *n* (%)	Men (*N* = 160) *n* (%)	Women (*N* = 322) *n* (%)	*P* value*
Non-CF at baseline	211 (43.8)	100 (62.5)	111 (34.5)	< 0.001
Non-CF	108 (51.2)	50 (50.0)	58 (52.3)	0.929
RCF	71 (33.6)	34 (34.0)	37 (33.3)	
PRCF	32 (15.2)	16 (16.0)	16 (14.4)	
RCF at baseline	219 (45.4)	46 (28.7)	173 (53.7)	
Non-CF	79 (36.1)	9 (19.6)	70 (40.5)	0.024
RCF	86 (39.3)	21 (45.7)	65 (37.6)	
PRCF	54 (24.7)	16 (34.8)	38 (22.0)	
PRCF at baseline	52 (10.8)	14 (8.8)	38 (11.8)	
Non-CF	24 (46.2)	9 (64.3)	15 (39.5)	0.101
RCF	13 (25.0)	4 (28.6)	9 (23.7)	
PRCF	15 (28.8)	1 (7.1)	14 (36.8)	

^*^All *p* values were assessed by Pearson’s Chi-squared test

**Fig. 2:**
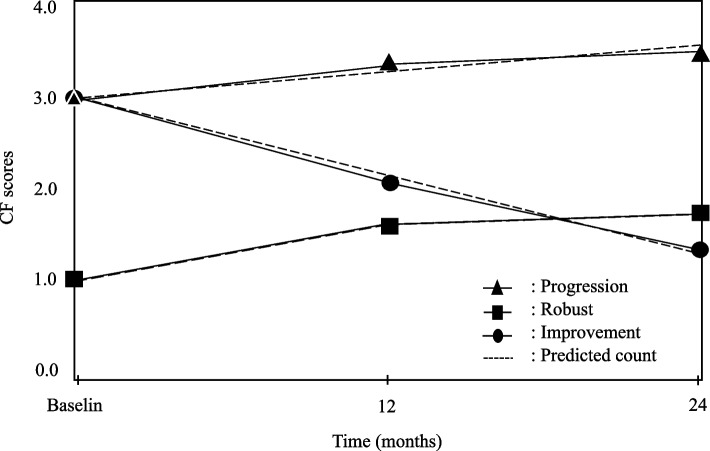
Plots of predicted counts (discontinuous lines) and observed values (solid lines with icons) from group-based trajectory models of cognitive frailty (CF) transitions at the baseline and two follow-up assessments. The three trajectory groups are progression (top line with triangles, *n* = 452, *p* < 0.001), improvement (middle line with dots, *n* = 142, *p* < 0.001), and robust (bottom line with squares, *n* = 238, *p* = 0.0320). Posterior probabilities of group membership were 0.82 for progression, 0.76 for improvement, and 0.78 for robust

Figure 2 illustrates the three CF trajectories selected from the group-based model. According to the model, 454 (54.6%) participants were classified as having CF progression, 142 (17.1%) as CF improvement, and 236 (28.4%) as CF robust; for this classification, the posterior probabilities of the three groups ranged 0.76 to 0.82. Furthermore, the sensitivity analysis displayed similar results of the three CF trajectories being most favorable for participants, with posterior probabilities of the three CF trajectories ranging 0.77 to 0.90.

Table 2 presents the distributions of baseline characteristics among the three CF trajectory groups of progression, improvement, and robust. The following variables significantly differed among the three groups: age, sex, BMI, educational level, monthly income, regular exercise habits, regular alcohol consumption of at least three times a week, the number of comorbidities, the number of medications, Tinetti gait, Tinetti balance, ADL scores, depressive status, ABC scores, MMSE scores, and gait characteristics of velocity, cadence, step width, stride length, and stride length variability. Conversely, no significant differences in the current smoking status or three gait variabilities of stride time, swing time, and double-support time were found.

**Table 2:** Comparisons of baseline characteristics among three cognitive frailty (CF) trajectory groups of stable, improvement, and progression

Characteristic	All **(***N* = 832) mean ± SD or *n* (%)	Robust **(***N* = 236) mean ± SD or *n* (%)	Improvement **(***N* = 142) mean ± SD or *n* (%)	Progression (*N* = 454) mean ± SD or *n* (%)	*P* value
Baseline CF state					
Non-CF	320 (38.5)	236 (100.0)	7 (4.9)	77 (17.0)	< 0.001
RCF	399 (48.0)	0 (0.0)	105 (73.9)	294 (64.8)	
PRCF	113 (13.6)	0 (0.0)	30 (21.1)	83 (18.3)	
Age, years	70.9 ± 5.1	69.7 ± 4.3	70.2 ± 4.4	71.7 ± 5.6	< 0.001
Sex					
Men	285 (34.3)	104 (44.1)	31 (21.8)	150 (33.0)	< 0.001
Women	547 (65.7)	132 (55.9)	111 (78.2)	304 (67.0)	
Body-mass index (kg/m^2^)					
Underweight	47 (5.6)	17 (7.2)	5 (3.5)	25 (5.5)	0.001
Normal weight	223 (26.8)	81 (34.3)	24 (16.9)	118 (26.0)	
Overweight	233 (28.0)	64 (27.1)	40 (28.2)	129 (28.4)	
Obese	329 (39.5)	74 (31.4)	73 (51.4)	182 (40.1)	
Educational level					
College or above	413 (49.6)	134 (56.8)	69 (48.6)	210 (46.3)	0.002
Senior and junior high	301 (36.2)	86 (36.4)	52 (36.6)	163 (35.9)	
Elementary school or lower	118 (14.2)	16 (6.8)	21 (14.8)	81 (17.8)	
Monthly income (NTD)					
Low (< 49,999)	415 (49.9)	82 (34.7)	68 (47.9)	265 (58.4)	< 0.001
Middle (50,000~99,999)	284 (34.1)	104 (44.1)	42 (29.6)	138 (30.4)	
High (≥ 100,000)	133 (16.0)	50 (21.2)	32 (22.5)	51 (11.2)	
Regular exercise (≥ 3 times per week)					
No	158 (19.0)	30 (12.7)	18 (12.7)	110 (24.2)	< 0.001
Yes	674 (81.0)	206 (87.3)	124 (87.3)	344 (75.8)	
Current smoking					
No	799 (96.0)	224 (94.9)	138 (97.2)	437 (96.3)	0.515
Yes	33 (4.0)	12 (5.1)	4 (2.8)	17 (3.7)	
Alcohol consumption					
No	735 (88.3)	195 (82.6)	129 (90.8)	411 (90.5)	0.005
Yes	97 (11.7)	41 (17.4)	13 (9.2)	43 (9.5)	
Number of comorbidities					
0 or 1	310 (37.3)	121 (51.3)	52 (36.6)	137 (30.2)	< 0.001
2 or 3	362 (43.5)	94 (39.8)	67 (47.2)	201 (44.3)	
≥ 4	160 (19.2)	21 (8.9)	23 (16.2)	116 (25.6)	
Number of medications					
0 or 1	169 (20.3)	73 (30.9)	31 (21.8)	65 (14.3)	< 0.001
2 or 3	411 (49.4)	121 (51.3)	73 (51.4)	217 (47.8)	
≥ 4	252 (30.3)	42 (17.8)	38 (26.8)	172 (37.9)	
Tinetti’s gait (0 ~ 13)	12.0 ± 1.4	12.4 ± 1.0	12.1 ± 1.1	11.8 ± 1.5	< 0.001
Tinetti’s balance (0 ~ 24)	23.2 ± 1.4	23.6 ± 0.8	23.2 ± 1.3	23.0 ± 1.6	< 0.001
ADL score					
28	810 (97.4)	233 (98.7)	141 (99.3)	436 (96.0)	0.032
< 28	22 (2.6)	3 (1.3)	1 (0.7)	18 (4.0)	
GDS score					
≤ 5	738 (88.7)	227 (96.2)	131 (92.3)	380 (83.7)	< 0.001
> 5	94 (11.3)	9 (3.8)	11 (7.7)	74 (16.3)	
ABC scale (0~100)	85.1 ± 11.3	89.7 ± 7.6	85.3 ± 11.7	82.7 ± 12.1	< 0.001
MMSE score	24.8 ± 2.2	25.5 ± 1.62	24.9 ± 2.3	24.3 ± 2.3	< 0.001
Gait characteristics					
Velocity (cm/s)	112.2 ± 22.1	120.7 ± 19.5	114.5 ± 20.3	107.0 ± 22.4	< 0.001
Cadence (steps/min)	111.0 ± 11.3	112.9 ± 10.4	113.1 ± 10.4	109.3 ± 11.7	< 0.001
Step width (cm)	61.5 ± 8.6	65.3 ± 7.4	61.7 ± 7.2	59.7 ± 8.2	< 0.001
Stride length (cm)	121.0 ± 16.8	128.5 ± 15.0	121.3 ± 14.9	117.0 ± 17.0	< 0.001
Stride length variability (%)	2.5 ± 2.1	2.1 ± 1.8	2.4 ± 2.1	2.7 ± 2.2	< 0.001
Stride time variability (%)	2.4 ± 1.6	2.2 ± 1.4	2.5 ± 1.7	2.5 ± 1.7	0.158
Swing time variability (%)	4.6 ± 3.5	4.2 ± 3.1	4.6 ± 3.7	4.8 ± 3.7	0.220
Double support time variability (%)	5.9 ± 3.8	5.5 ± 3.8	6.1 ± 3.8	6.0 ± 3.7	0.167

*ABC* Activities-specific Balance Confidence, *ADLs* Activities of daily living, *CF* Cognitive frailty, *GDS* Geriatric Depression Scale, *MCI* Mild cognitive impairment, *MMSE* Mini-Mental Status Examination, *NTD* New Taiwan dollar (the approximate exchange rate in 2021 was US$1≈NTD30), *PRCF* Potentially reversible cognitive frailty, *RCF* Reversible cognitive frailty, *SD* Standard deviation

Table 3 presents results of the multivariable multinomial logistic regression analysis of age, sex, and other explanatory variables for CF progression and CF improvement versus CF robust over the study period. In model 1, after adjusting for the baseline CF state, each 1-year increase in age significantly increased the odds of CF progression by 8% (OR = 1.08; 95% CI, 1.02 ~ 1.14). Each 1-point increase in Tinetti balance scores significantly reduced the odds of CF progression by 28% (OR = 0.72; 95% CI, 0.54 ~ 0.96), and each 1-cm/s increase in gait velocity significantly reduced the odds of CF progression by 2% (OR = 0.98; 95% CI, 0.97 ~ 0.99). Participants with four or more comorbidities had a significantly increased odds of CF progression by 165% (OR = 2.65; 95% CI, 1.19 ~ 5.90), compared to those with one comorbidity/no comorbidities. CF improvement was not significantly associated with any variable except the baseline CF state. In model 2, without adjusting for the baseline CF state, an older age, female sex, depression, and a higher number of comorbidities were significantly associated with an increased odds of CF progression, while engaging in regular exercise, having a higher Tinetti balance score, and having a faster gait velocity were significantly associated with a reduced odds of CF progression. Female sex and a higher number of comorbidities were significantly associated with an increased odds of CF improvement, while a higher Tinetti balance score was significantly associated with a reduced odds of CF improvement.

**Table 3:** Results of the multivariable multinomial logistic regression of explanatory variables with odd ratios (ORs) and 95% confidence intervals (CIs) for cognitive frailty (CF) improvement and CF progression vs. CF robust

Characteristic	Model 1				Model 2			
	Improvement OR (95% CI)	*p* value	Progression OR (95% CI)	*p* value	Improvement OR (95% CI)	*p* value	Progression OR (95% CI)	*p* value
Baseline CF state								
Non-CF	1.00 (reference)		1.00 (reference)		---	---	---	---
RCF	3.6 × 10^3^ (4.3 × 10^2^ ~ 3.0 × 10^4^)	< 0.001	9.7 × 10^2^ (1.3 × 10^2^ ~ 7.2 × 10^3^)	< 0.001	---	---	---	---
PRCF	8.4 × 10^2^ (9.9 × 10 ~ 7.1 × 10^3^)	< 0.001	1.8 × 10^2^ (2.4 × 10 ~ 1.3 × 10^3^)	< 0.001	---	---	---	---
Age (years)	1.05 (0.98 ~ 1.12)	0.141	1.08 (1.02 ~ 1.14)	0.004	1.02 (0.97 ~ 1.08)	0.348	1.06 (1.02 ~ 1.11)	0.002
Women (vs. men)	1.11 (0.56 ~ 2.20)	0.770	0.72 (0.42 ~ 1.23)	0.226	3.07 (1.87 ~ 5.04)	< 0.001	1.63 (1.13 ~ 2.35)	0.009
Tinetti balance (0 ~ 24)	0.77 (0.56 ~ 1.06)	0.106	0.72 (0.54 ~ 0.96)	0.027	0.70 (0.56 ~ 0.89)	0.003	0.69 (0.56 ~ 0.85)	< 0.001
Gait velocity (cm/s)	1.00 (0.98 ~ 1.02)	0.984	0.98 (0.97 ~ 0.99)	0.044	0.99 (0.98 ~ 1.01)	0.307	0.98 (0.97 ~ 0.99)	< 0.001
Number of comorbidities								
0 or 1	1.00 (reference)		1.00 (reference)		1.00 (reference)		1.00 (reference)	
2 or 3	1.64 (0.81 ~ 3.32)	0.170	1.54 (0.86 ~ 2.76)	0.150	1.78 (1.10 ~ 2.85)	0.018	1.62 (1.11 ~ 2.36)	0.012
≥ 4	1.82 (0.69 ~ 4.76)	0.224	2.65 (1.19 ~ 5.90)	0.017	2.15 (1.06 ~ 4.37)	0.034	2.90 (1.65 ~ 5.10)	< 0.001
Regular exercise (≥ 3 vs. < 3 times per week)	---	---	---	---	1.29 (0.68 ~ 2.47)	0.439	0.58 (0.36 ~ 0.94)	0.028
GDS score (> 5 vs. ≤ 5)	---	---	---	---	1.67 (0.67 ~ 4.17)	0.270	3.10 (1.50 ~ 6.41)	0.002

*GDS* Geriatric Depression Scale, *PRCF* Potentially reversible cognitive frailty, *RCF* Reversible cognitive frailty

## Discussion

To our knowledge, this is the first study to identify latent CF trajectories using a group-based model approach, in which three CF trajectory groups of progression, improvement, and robust were recognized. We observed that individuals with CF progression and those with CF improvement had similar baseline CF states, and the two trajectory groups displayed higher baseline CF scores vs. the robust group, so that the baseline CF state was adjusted for in model 1. It should be noted that the predictors in model 1 may have explained the progression and improvement of CF during the study period, while those in model 2 (without adjustment for the baseline CF state) may have explained the progression and improvement of CF during the study period and before the study period, since the baseline CF state could reflect the development of CF before the study began [36]. Specifically, the three variables of female sex, regular exercise, and depression, which were only statistically significant in model 2 but not in model 1, might have only contributed to the baseline CF state but their contribution to CF progression or CF improvement during the study was ambiguous.

An older age was significantly associated with CF progression but not with CF improvement, implying that the aging process at the biological, psychological, social, and environmental levels could monotonically increase levels of CF in older populations. CF may represent a state of age-related decline in brain neurophysiological reserves that is related to features of both neurodegenerative disorders and vascular diseases [37]. Several studies reported that age-related neuropathologies might have adverse effects on both physical frailty and cognitive decline [38, 39], while most studies found that physical frailty occurred earlier than cognitive decline [40, 41], and some demonstrated that cognitive impairment should lead to subsequent frailty [42, 43]. Nonetheless, common pathological mechanisms underlying cognitive frailty still remain unclear, and acquiring more knowledge of interactions between physical function and the cognitive status may help clarify the underlying mechanisms of CF.

In this study, higher Tinetti balance scores were significantly associated with a lower risk of CF progression. Prior studies also found lower scores of the timed up and go test and functional reach in frail older adults [44]. Poor balance was found to be associated with physical measures that include slow gait velocity, exhaustion, a high number of comorbidities, and falls history [45] and cognitive measures that include lower cognitive function and faster cognitive declines in global cognition, episodic memory, and processing speed [46, 47]. Levels of balance control were reduced with an increasing severity of cognitive impairment, particularly in executive functioning, for which among patients with MCI, mild AD, and moderate AD, those with moderate AD exhibited the worst balance performance [48]. However, knowledge of physiopathological mechanisms underlying the relationship between balance ability and CF or cognitive decline remain to be explored.

A higher number of comorbidities was significantly associated with CF progression in this study. Prior studies reported that the coexistence of multiple chronic conditions had impacts on either motor functions (e.g., gait velocity and ADL) or cognitive performance [49, 50], rather than on both simultaneously. Meta-analytical results showed that approximately 16% of those with comorbidities also presented with frailty; in turn, 72% of frail people presented with comorbidities [51]. On the other hand, the number of comorbidities also increases with severity levels of cognitive impairment [52], and a higher number of comorbidities may rapidly increase the risk of progression to dementia [53]. Similar to many developed countries, the most common chronic conditions in Taiwan (e.g., hypertension, diabetes, heart disease, and high cholesterol) [50] are also vascular-related risk factors that may contribute to both cognitive decline and physical frailty and eventually exert detrimental effects on every organ [54].

Gait velocity was significantly associated with CF progression in this study. While gait velocity has been a powerful predictor of adverse outcomes, such as disability, falls, hospitalizations, and mortality [55], a decline in gait velocity may also predict AD and non-AD dementia and even precedes the decline in cognitive performance by 5 years among healthy older adults [56]. A growing number of neuroimaging studies support that gait and cognitive functions share brain areas and networks, particularly in the prefrontal cortex and hippocampus [57]; for instance, smaller cortical gray matter volumes and smaller hippocampal volumes were associated with a slower gait, and these associations were weakened by controlling for cognitive performance [58]. Therefore, gait characteristics might elucidate a common basic mechanism of cognitive and motor declines [59].

Three factors of female sex, engaging in regular exercise, and depression were statistically significant in model 2 but not in model 1, indicating they could affect the baseline CF state (or CF development before the study) but did not significantly contribute to the progression or improvement of CF during the study period. Possible reasons for the sex difference in CF might be variance in sex hormone reductions (androgens and estrogens), genetic risks (e.g., apolipoprotein E epsilon 4 allele), brain structure, volum, and glucose metabolism, and some diseases (e.g., diabetes and cardiovascular diseases) [60]. In the present study, regular physical exercise was independently associated with CF progression but not with CF improvement. Reduced physical activity can be a precursor for cardiometabolic diseases, such as unhealthy weight gain and obesity, high cholesterol, hypertension, and insulin resistance, which may contribute to cognitive impairment and incident frailty [61]. Potential mechanisms through which exercise training improves cognitive function might include increases in cerebral blood flow and neurotrophic factors (e.g., brain-derived neurotrophic factor), downregulation of neurotoxic factors (e.g., C-reactive protein) and other inflammatory cytokines, better control of chronic diseases, and prevention of depression [62]. Depression may result in muscle weakness, reduced physical activity, feelings of fatigue, and a slow gait velocity, and thereby be linked to physical frailty [50], which may also increase the risk of cognitive impairment [63], particularly in information processing and working memory deficits [64]. Alternatively, the occurrence of physical frailty may be enhanced due to late-life depression even after controlling for comorbidities and disabilities [65]. The three factors of female sex, regular exercise, and depression might only contribute to the baseline CF state; hence, any inference of causal relationships between these factors and CF development should be made with caution.

There are several limitations in this study. First, the score assigned to each CF state was somewhat arbitrary, and its scalability needs to be psychometrically validated, since differences in scores between two adjacent CF states were presumed to be the same. Second, we made use of data on all participants in the group-based trajectory model, and those who missed one or two follow-up assessments tended to have PRCF or RCF. Although the sensitivity analysis identifying the latent CF trajectory groups based on participants who completed the two follow-ups showed similar findings, our results might be somewhat biased due to loss of follow-up. Furthermore, the size or probability of CF progression might have been further underestimated due to participants who died during this study being excluded. Third, while not all health conditions were assessed in this study or health interventions other than regular exercise habits and number of medications for the participants were not included, unmeasured health conditions and interventions might have had an impact on the longitudinal patterns of CF transitions. Finally, the study period was short. It is possible that our data from two follow-ups might have been insufficient to estimate stable profiles of the CF trajectory, and the number of CF trajectories based on the group-based model might change if more data points were obtained.

## Conclusions

This study demonstrated that the baseline CF state, an older age, poorer balance, a slower gait velocity, and a high number of comorbidities may contribute to CF progression, while only the baseline CF state was associated with CF improvement. Despite engaging in regular exercise and depressive symptoms being associated with CF progression, their associations could be accounted for by the baseline CF state. Further investigations with a longer follow-up period are needed to confirm our results.

### Supplementary Information

**Additional file 1: Supplementary Table 1.** Score assigned for each of the cognitive-frailty (CF) states.

## Data Availability

The datasets generated and analyzed during the current study are not publicly available due to ethical restrictions and patient confidentiality but are available from the corresponding author upon reasonable request.

## Abbreviations

ABC: Activities-specific Balance Confidence
AD: Alzheimer’s disease
ADLs: Activities of daily living
BMI: Body-mass index
CDR: Clinical Dementia Rating
CF: Cognitive frailty
MCI: Mild cognitive impairment
MMSE: Mini-mental state examination
PRCF: Potentially reversible cognitive frailty
RCF: Reversible cognitive frailty
