# Supplementary material for: Factors affecting cognitive frailty improvement and progression in Taiwanese older adults
Source: BMC Geriatr. 2024 Jan 29;24:105. doi: 10.1186/s12877-024-04700-3 (PMC10823623; doi:10.1186/s12877-024-04700-3)
Supplement: Supplementary file 1 — Additional file 1: Supplementary Table 1. Score assigned for each of the cognitive-frailty (CF) states. [file 12877_2024_4700_MOESM1_ESM.docx]

Supplementary Table 1. Score assigned for each of the cognitive-frailty (CF) states

| Characteristics of the CF state | Score |
| --- | --- |
| Robust (non-frailty + no SCD) | 0 |
| Presence of pre-frailty or SCD only | 1 |
| Presence of frailty or MCI only | 2 |
| Simultaneous presence of pre-frailty and SCD | 3 |
| Simultaneous presence of pre-frailty and MCI or frailty and SCD | 4 |
| Simultaneous presence of frailty and MCI only | 5 |

MCI, mild cognitive impairment; SCD, subjective cognitive decline.
